# Protective Role of *Rheum Tanguticum *Polysaccharide 1 in Radiation- induced Intestinal Mucosal Injury

**Published:** 2015

**Authors:** Lin-Na Liu, Lei Shi, Shi-Cao Li, Wen-Juan Zhang, Yan Zhang, Zhi-Pei Zhang

**Affiliations:** a *Pharmacy Department, Tangdu Hospital, Fourth Military Medical University, Xi’an, China.*; b *Laboratory of Department of Thoracic Surgery, Tangdu Hospital, Fourth Military Medical University, Xi’an, China.*

**Keywords:** Polysaccharide, *Rheum tanguticum*, Ionising radiation, Small intestine, Radioprotection

## Abstract

The protective effects of Rheum tanguticum polysaccharide 1 (RTP1), which is extracted from the Chinese traditional medicine *Rheum tanguticum*, on radiation-induced intestinal mucosal injury was investigated. Rat intestinal crypt epithelial cells (IEC-6 cells) and Sprague-Dawley rats were each divided into control, irradiated and RTP1-pretreated irradiated groups. After irradiation, cell survival was determined by MTT (3-[4,5-dimethylthiazol-2-yl]-2,5 diphenyl tetrazolium bromide). assay, and the intracellular reactive oxygen species (ROS) was detected by fluorescent probe method. Apoptosis was observed by acridine orange staining, and cell cycle was analysed by flow cytometry. Histological analysis of the rat intestinal mucosa was conducted by haematoxylin and eosin staining. Irradiation at 8 Gy(Gray) decreased cell survival rate to only 54%, significantly increased intracellular ROS levels and induced apoptosis. RTP1 pretreatment significantly inhibited cell death, reduced the formation of intracellular ROS and partially inhibited apoptosis. Irradiation markedly reduced the height and quantity of rat intestinal villi, but it could be antagonised by RTP1 pretreatment. RTP1 can promote the recovery of intestinal mucosa damage, possibly by inhibiting radiation-induced intestinal epithelial apoptosis and intracellular ROS production.

## Introduction

Radiation therapy is an inevitable choice for most cancer patients, especially for those with abdominal and pelvic tumours. However, this therapy causes radiation enteritis because the gut is an important target organ of radiation damage, which is one of the most difficult complications in abdominal tumour radiotherapy (1-4). The incidence of acute radiation enterocolitis is reportedly 50% to 70%. Radiation injury to the gut occurs during radiotherapy and can last up to 10 years after treatment in some populations, with an incidence of approximately 5% to 10% (5,6). An increasing body of evidence shows that injury in the intestinal mucosa may further aggravate the primary disease and induce multiple organ dysfunction and systemic inflammatory response syndrome, leading to a vicious and possibly life-threatening circle (7,8). The treatment for radiation-induced intestinal damage is currently mainly based on symptomatic and supportive (anti-diarrheal, analgesic and anti-inflammatory) treatment or through surgical, hyperbaric oxygen and nutritional support approaches. However, all these methods exhibit little ideal therapeutic effect and lack the use of preventive drugs (9). Therefore, developing new methods with preventive effect on radiation damage and can promote the recovery of radiation-induced intestinal mucosal injury is of great significance. Searching for effective substances in natural products is now an important step in protecting the gut from radiation injury.

*Rheum tanguticum* Maxim ex Reg. is a Chinese traditional medicine. Water-soluble polysaccharides were extracted from *R. tanguticum* and separated by gel column chromatography. Polysaccharides with five different molecular weight fractions (RTP1 to RTP5) were obtained. Animal experiments showed that RTP1 (molecular weight of 6 × 10^5 ^to 8 × 10^5^) can significantly reduce the mortality of rats with 2,4,6-trinitrobenzene sulphonic acid-induced colitis by reducing the weight of the colon, thereby narrowing the ulcer area and relieving mucosal oedema (10). RTP1 pretreatment can reduce hydrogen peroxide-induced intestinal epithelial cell apoptosis, enhance cell viability, increase superoxide dismutase (SOD) activity and decrease malondialdehyde (MDA) level as well as lactate dehydrogenase (LDH) activity. This anti-oxidative damage process is related to reductions in the pro-apoptotic gene Bax and Caspase-3 activity (11). The present study was conducted to investigate the protective role of RTP1 in radiation-induced intestinal epithelial cell injury and provide a basis for exploring new drugs with protective effects on radiation-induced intestinal injury.

## Experimental

*RTP1* *preparation *

Rhubarb crude polysaccharide was extracted by water extraction and alcohol precipitation. Crude polysaccharide was then separated and purified using DEAE-52 column (Shanghai Rego Biotechnology Co., *Ltd*., Shanghai, China) chromatography and Sephacryl S-200 gel column (GE China-Healthcare, Beijing, China) chromatography. Polysaccharides with five different molecular weight fractions (RTP1 to RTP5) were obtained. RTP1 was used in this experiment and dissolved with phosphate buffer solution (PBS, pH 7.4) prior to use.

*Cell culture*

IEC-6 cells (rat small intestinal epithelial cell line CRL-1592, 14 passage was used in the experiment) from American Type Culture Collection (Rockville, MD, USA) were grown in RPMI1640 complete medium supplemented with 10% fetal bovine serum (FBS), 100 IU/mL penicillin and 100 μg/mL streptomycin (Invitrogen-Gibco, Carlsbad, CA, USA) and then incubated in an incubator under 5% CO_2_ at 37 °C. The culture medium was changed every 2 or 3 d. The cells were passaged as they grew to 80% confluence.

*Cell viability determination*

The IEC-6 cells were divided into the control (NC), irradiated (IR) and RTP1-pretreated irradiated (RTP1+IR) groups. The control cells were cultured normally without any treatment. The IR and RTP1+IR groups were exposed to 8 Gy radiation (Varian Clinac 23EX, Varian medical systems, Salt Lake City, USA). The RTP1+IR group was pretreated with 10 μg/mL RTP1 2 h prior to irradiation. All cells from the three groups were then incubated under 5% CO_2_ at 37 °C for 48 h. Thiazolyl blue was added to the cells to a final concentration of 5 mg/mL (MTT, Sigma-Aldrich, St. Louis, MO. USA) and incubated for an additional 4 h. Dimethyl sulfoxide (DMSO) was added to dissolve the insoluble purple formazan product into a coloured solution. The absorbance was quantified using a spectrophotometer (BioRad, Philadelphia, PA, USA) at 570 nm. Each group was replicated in at least six wells.

*Intercellular ROS* *measurement*

The level of intracellular reactive oxygen species (ROS) was measured using the fluorescent probe agent 2,7-dichlorodihydrofluorescein diacetate (Molecular Probes, Eugene, Oregon, USA), which can enter into cells with the help of acetoacetate. After hydrolysis, oxidisation and dehydrogenisation in cell, fluorescein becomes luminescent, and its luminous intensity is proportional to the level of intracellular ROS. Thus, fluorescence intensity can reflect ROS level. IEC-6 cells were seeded into dedicated confocal small dishes at a density of 1 × 10^3^ cells/ml and then grouped and treated as described above. Intercellular ROS level was determined at 48 h post-irradiation using fluorescent probe methods, as previously described (12). The cells were washed twice with PBS, and 100 mL fluorescent probe supporting liquid 2,7-Dichlorodihydrofluorescein diacetate (DCFH-DA,10 mmol/L) was then added. After incubation at 37 °C for 30 min in the dark, intracellular ROS was measured using a laser confocal microscope at an excitation wavelength of 488 nm and a detection wavelength of 530 nm to 560 nm. After background calibration, the obtained image was analysed using Zeiss software (Carl Zeiss Meditec, Suzhou, China) to calculate the fluorescence intensity per square millimetre of cell area.

*Apoptosis* *detection*

Acridine orange staining was used as a morphological indicator to observe the apoptosis of the irradiated IEC-6 cells. The cells were cultured on poly-L-lysine-treated coverslips, grouped and treated as described in Section 2.3. After experimentation, the coverslips were quickly rinsed with warm PBS for 3 s to 5 s to remove the FBS from the medium and then placed in 95% ethanol. After being fixed in 95% ethanol for 15 min, the coverslips were retrieved, and the excess liquid was removed. The coverslips were then treated with 1% acetic acid for 30 s, stained with 2 μg/mL acridine orange for 1 min, treated with 0.1 M CaCl_2_ for 30 s, rinsed thrice with PBS and then observed under a fluorescence microscope with a blue-green filter. Changes in the nuclei of the IEC-6 cells were observed and photographed. The apoptosis of IEC-6 cells were then qualitatively observed.

*Cell cycle detection*

IEC-6 cells in the logarithmic growth phase were harvested and seeded in culture flasks at a density of 2 × 10^6^ cells per flask. After allowing the cells to adhere, the supernatant was removed, and the cells were grouped and treated as described in Section 2.3. After experimentation, the cells were harvested and washed once with cold PBS (0.1 M). Then, the cells were resuspended in 1 mL PBS and fixed with 2 mL dehydrated alcohol for 30 min. Afterwards, all cells were collected and washed once with PBS and then stained with 50 μg/mL propidium iodide (Boehringer Mannheim, Indianapolis, USA) in the dark for 30 min at room temperature. The cells were then analysed using flow cytometry (Coulter XL, Beckman Coulter, Inc., Fullerton, CA, USA) to determine apoptosis and cell cycle distribution. All tests were performed in triplicate. 

*Sprague-Dawley rats treated with RTP1*

Male and female Sprague-Dawley rats, weighing 200 ± 20 g and with certification number SCXK 20022010, were obtained from the Experimental Animal Centre of the Fourth Military Medical University (Xi’an, Shaanxi, PR China). *This study was conducted in strict compliance with the Guide for the Care and Use of Laboratory Animals of the National Institutes of Health. The animal use protocol was reviewed and approved by *the Institutional Animal Care and Use Committee of Fourth Military Medical University. All animals were housed separately at a constant temperature of 23 ± 3 °C and a relative humidity of 45% to 55% with 12 h light per day. The rats had free access to water and food. A total of 18 rats were equally divided into 3 groups randomly: control (NC), physiological saline-pretreated irradiated (IR) and the RTP1-pretreated irradiated (IR+RTP1) groups. The rats in the IR+RTP1 group were intraperitoneally injected with 10 mg/Kg RTP1 for 14 d. The rats in the NC and IR groups were treated with the same dose of physiological saline in parallel. On day 14, the rats in the IR and IR+RTP1 groups underwent disposable systemic uniform irradiation of 8.0 Gy at a distance of 8 °Cm (13). Four days after irradiation, the rats were anesthetised and killed, after which 2 cm of small intestine 1 °Cm away from the Treitz ligament was removed and instantly fixed in 40 g/L neutral formalin. Following the conventional procedure, the tissues were embedded in paraffin, cut into 6 μm slices, adhered onto the slides, stained with haematoxylin and eosin and observed under a light microscope. Three non-continuous slices were analysed using a Leica image analyser (Leica Microsystems Ltd., Hong Kong, China) with completely blind testing. The numbers and heights of villi within 10 visual fields were recorded. The height of the villi was measured from the base of a long villus to the top, and the average height was recorded (14).

*Statistical analysis*

Cell viability data are shown as percentages and compared using χ^2^-test. Other data are presented as mean ± standard deviation and analysed using SPSS software v10.0 (Chicago, IL, USA). Differences among groups were compared using one-way ANOVA. Pairwise comparison between groups was performed using the *q*-test. A p-value < 0.05 was considered statistically significant.

## Results

*Effects of RTP1 on radiation-induced injury*

**Figure 1:**
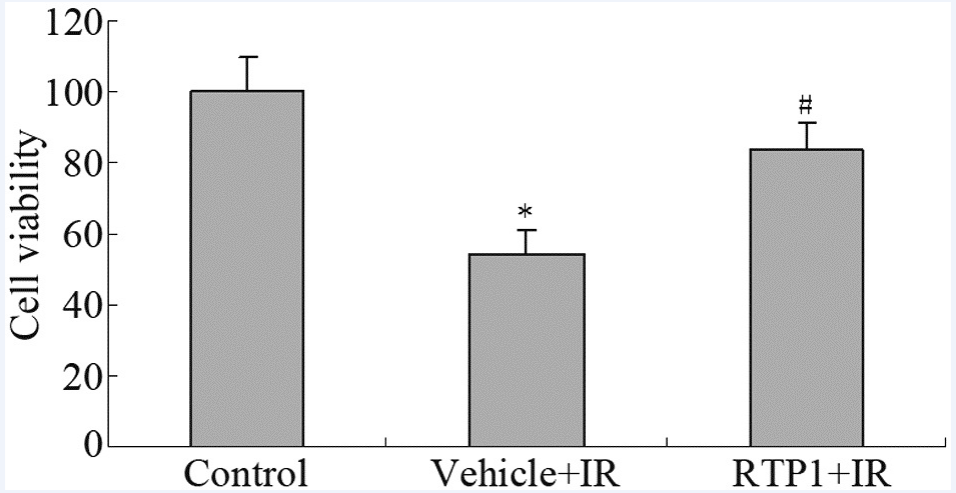
Effect of RTP1 on cell viability. *P < 0.05 *vs* control; ^#^*P* < 0.05 *vs* Vehicle+IR.

The results of the MTT assay show that 8 Gy radiation reduced the survival rate of IEC-6 cells to only 54%. However, RTP1 pretreatment significantly inhibited the radiation-induced cell death of IEC-6 cells. The cell survival rate was restored to 83% (Figure 1).

*Effects of RTP1 on ROS level*

**Figure 2:**
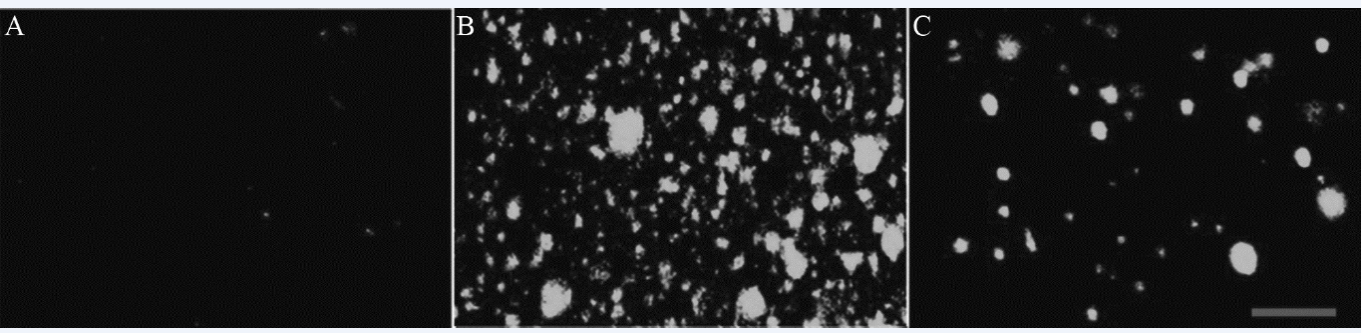
Laser confocal scanning microscope image of DCF fluorescence in cells of different groups. A: Control; B: Vehicle+IR; C: RTP_1 _+IR.

Under 8 Gy irradiation, the intracellular ROS level of IEC-6 increased dramatically, as indicated by the significant increase in fluorescence intensity. However, the ROS increase was antagonised by RTP1. The fluorescence intensity was reduced to 54.1% (Figure 2 and Table 1).

**Table 1:** Effect of RTP1 on the intracellular ROS in the irradiated IEC-6 cells (*n* = 6).

**Group**	**DCF fluorescence intensity**
Control	10.3 ± 2.8
Vehicle+IR	93.2 ± 10.5**
RTP_1 _+IR	39.1 ± 8.7##

Data are shown as mean±standard deviation (SD);

** *P *< 0.01 *vs* control;

## *P *< 0.01 *vs* Vehicle+IR

*Effects of RTP1 on apoptosis*

**Figure 3:**
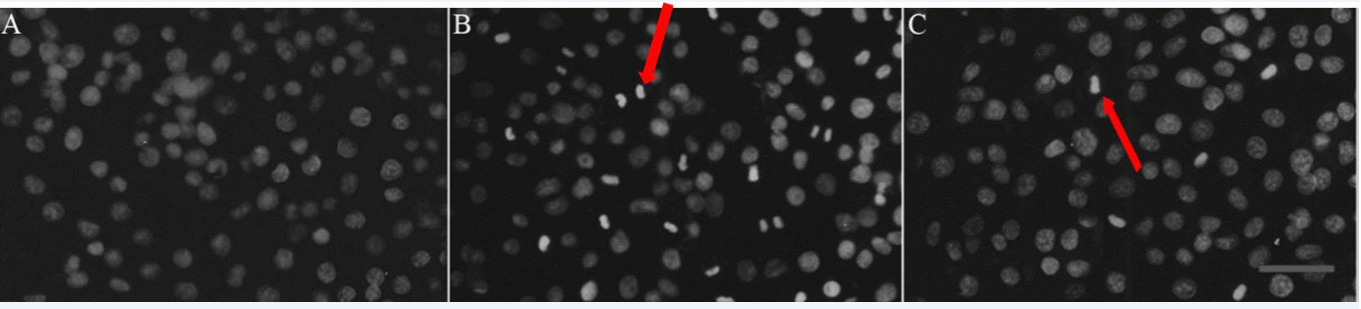
Effect of RTP_1_ on IEC-6 cell apoptosis identified by acridine orange staining.

At 48 h post-irradiation with 8 Gy ray, the cells were stained with acridine orange and observed under a fluorescence microscope. The nuclei of the IEC-6 cells in the control group were normal, which exhibited a dispersed, uniform green fluorescence. However, in the IR group, numerous typical apoptotic cells that contained condensed cytoplasm and seemingly hyperchromatic nuclei appeared, dense bright green fluorescence was observed and apoptotic bodies were found. RTP1 pretreatment partially suppressed apoptosis. The number of apoptotic cells in the RTP1+IR group was less than that in the IR group (Figure 3). Thus, RPT1 pretreatment can significantly reduce radiation-induced apoptosis of intestinal epithelial cells.

A: Control; Cells in normal group showed normal nuclei size, shape and greenish-structure with different shades. B: Vehicle+IR; After exposure to radiation, typical morphological characteristics of apoptosis were identified. Apoptotic cells demonstrated chromatin aggregation in compact masses and strong green fluorescence in the nuclear (as shown at the red arrow). C: RTP1 +IR. Pretreatment with RTP1 could partially inhibit cell apoptosis (as shown at the arrow).

*Effects of RTP1 on cell cycle*

**Figure 4:**
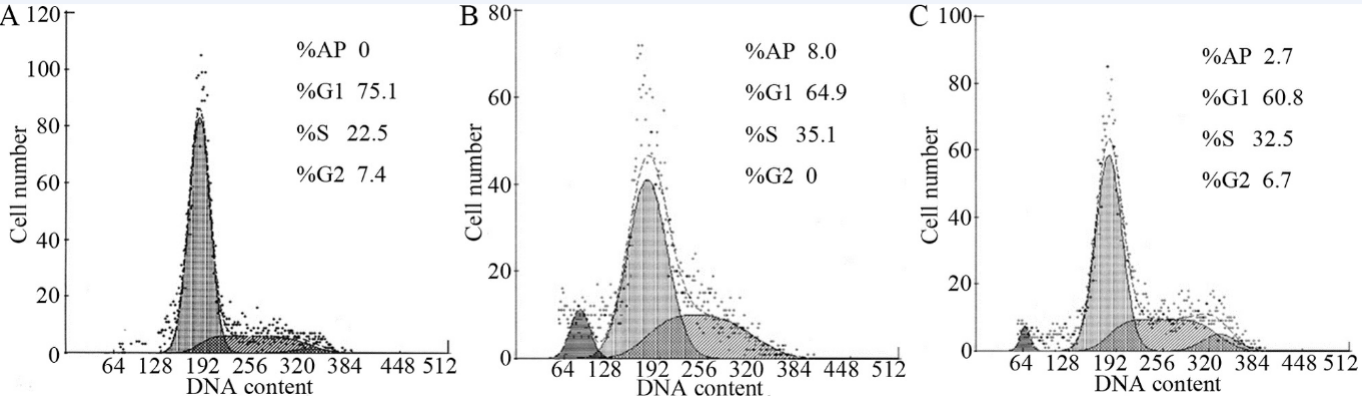
Effects of RTP1 on IEC-6 cell cycle progression. A: Control; B: Vehicle+IR; C: RTP_1 _+IR.

The hypodiploid peak that appeared before the G1 peak was the apoptotic peak formed by the apoptotic cells and the characteristic result of apoptotic cell pyknosis and DNA cleavage. Flow cytometry results show the absence of an apoptotic peak in the IEC-6 cells of the control group, whereas an apoptotic peak ratio of (8.0±0.67)% was observed in the IR group. Upon RTP1 pretreatment, the apoptotic peak proportion decreased to (2.7±0.26)%. In the IR group, the cell proportion in the S phase increased remarkably and those in the G0/G1 and G2/M phases decreased correspondingly, suggesting that the cells were arrested in the S phase (Figure 4).

*Effects of RTP1 on rats*

**Figure 5:**
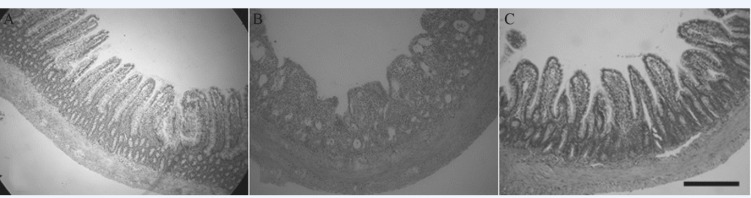
Representative images showing height of villi and crypt cell survival in the intestinal circumference of different groups. A: Control; B: Physiological saline+IR; C: RTP_1 _+IR.

To investigate the effects of RTP1 on the radiation-induced intestinal mucosa injury *in-vivo*, we created a rat model exhibiting radiation-induced intestinal mucosa injury and an RTP1-pretreated model. We found that all the rats in the IR group suffered from diarrhoea without loose stools that contained large amounts of water and mucus. Moreover, in the IR group, the rat’s food intake were reduced, the rats’ clear eyes became dull, their coat lost luster, some fur flaked off the coat, their spirit flagged and accompanied with weight loss. By contrast, rats in the control group grew gradually. The rats in IR+RTP1 group also suffered from diarrhoea during the first 2 d post-irradiation, but it was ameliorated on day 3 post-irradiation. The stools of the rats in the IR+RTP1 group contained less mucus than those in the IR group. However, the stools of the former were still wetter and softer than those in the control group. The anatomy of the rats showed that the bowel in the control group was pink without any congestion, oedema or volume expansion. Histological analysis showed that the rats in the control group had neat and compact mucosa villi, and their small intestinal villi had a clear outline, complete surface structure and large villus height and crypt depth. The intestinal canal of the rats in the IR group was dark red, had an expanded volume and was congested. The intestinal wall became thin because of mucosal bleeding, ulceration and perforation. Histological analysis showed that the small intestinal mucosa epithelium of the rats in the IR group was stripped, shortened, disorderly and necrotic. The surface structure of the villi was incomplete and extensively damaged. In the RTP1+IR group, the intestinal mucosa was obviously better than that in the IR group. Volume expansion and intestinal mucosal hyperaemia were clearly reduced. Under a light microscope, the small intestinal mucosal villi were arranged more neatly and orderly, and the nap shrunk more slightly compared with that in the IR group (Figure 5). The heights and number of the small intestinal villi of the rats in the three groups are listed in Table 2, which shows that the heights and number of the small intestinal villi of the rats were reduced significantly in the IR group (*P *< 0.01 or 0.05), compared with the control group. However, the heights and width of the villi decreased slightly in the RTP1+IR group. Moreover, the heights of the villi of the rats in the RTP1+IR group were not significantly different from those in the control group, which were much higher than those in the IR group (*P *< 0.01 or 0.05).

**Table 2:** Number and height of villi in the three groups (*n* = 6).

**Group**	**Villous number**	**Villous height (mm)**
Control	9.5 ± 0.8	0.499 ± 0.033
Vehicle+IR	3.7 ± 0.7**	0.149 ± 0.028**
RTP1+Irradiation	8.0 ± 0.93##	0.389 ± 0.039#

Data are shown as mean±SD;

** *P *< 0.01 *vs* control;

# *P *< 0.05 *vs* Vehicle+IR,

## *P *< 0.01 *vs* Vehicle+IR

## Discussion

The intestine is the abdominal organ that is most sensitive to radiation (15) and extremely vulnerable to injury and acute radiation enteritis and other complications during the radiotherapy of basin abdominal tumour. This sensitivity seriously affects the effects of therapies as well as the quality of patients’ lives. No specific drug that prevents and treats intestinal radiation injury is currently available (1-4,9,16). Therefore, formulating a substance with protective effect against intestinal radiation injury is clinically important.

Our previous studies have shown that RTP1 has obvious protective effect on the stress gastric ulcer in rats caused by water bondage stress. RTP1 can also clearly reduce the mortality of rats with colitis induced by 2,4,6- trinitro-benzene-sulphonic acid, reduce colon weight, deflate colon ulcer area and alleviate mucosal oedema. *In-vitro* studies have shown that in the IEC-6 of normal rat small intestinal crypt cells, RTP1 can increase ornithine decarboxylase activity and protein expression by removing polyamine, thereby promoting the proliferation, migration and differentiation of intestinal epithelial cells. RPT1 pretreatment can reduce radiation-induced enterocyte apoptosis and enhance cell viability. Moreover, this pretreatment can increase SOD activity as well as decrease MDA level and LDH activity. This anti-oxidative process is related to the decrease in the activities of pro-apoptosis gene Bax and Caspase 3 (10,11). These results suggest that RTP1 can promote the repair of intestinal mucosa injury, which is associated with inhibition of the apoptosis of intestinal epithelial cells, resistance to oxidation damage and immune regulation. Given that radiation can also cause apoptosis and oxidative damage on intestinal mucosa (17,18), we therefore observed the effects of RTP1 on acute radiation-induced injury of intestine in this study.

Results show that radiation can inhibit the normal proliferation of the IEC-6 of rat small intestinal crypt epithelial cells. However, this inhibition can be resisted by RTP1 pretreatment, thereby increasing the cell survival rate. Moreover, RTP1 can inhibit the radiation-induced apoptosis of intestinal epithelial cells, suggesting that RTP1 reduces the intestinal epithelial cell death caused by radiation by inhibiting the apoptosis of intestinal epithelial cells. Apoptosis plays an important role in maintaining the steady state of intestinal mucosa epithelial cells and is important in maintaining the cell number balance (18). Apoptosis can occur after stimulations such as ischemia, hypoxia and physical and chemical damage. Various studies have shown that intestinal cell apoptosis increases significantly after exposure to radiation, which may be related to radiation-induced ischemia, hypoxia, large production of oxygen free radicals and inflammatory mediators, inhibition of mitosis and direct damage to DNA, and so on (19,20). The free radical theory on radiation injury also suggests that free radicals and ROS produced by hydrolysis are important factors that result in radiation injury. Large amount of ROS can destroy biological macromolecules, such as DNA and plasma membrane, and further cause cellular damage, death and gene mutation. Thus, reducing ROS generation post-irradiation and accelerating ROS removal in tissues and cells are important functions of radiation-protective medicine (19,20). This study shows that although radiation can increase the amount of intracellular reactive oxygen, this increase can be significantly reduced by RPT1 pretreatment, suggesting that RTP1 plays a role in clearing intracellular oxygen free radicals. The analysis on cell cycle indicates that radiation damage can increase the proportion of S phase cells and reduce the proportion of G0/G1 phase cells in the IR group, and that RTP1 pretreatment can improve the proportion of S phase cells and increase the proportion of G0/G1 and G2/M phase cells. The total numbers of S and G2/M phase cells reflect cell proliferation ability, which indicates that RTP1 may inhibit the radiation-induced apoptosis of intestinal epithelial cells and reduce intestinal epithelial cell death by reducing intracellular ROS.

Animal experiments in this study also support the above conclusion. The height and width of small intestinal mucosal villi determine the villus surface area, and thus affects digestion and absorption function. Villus epithelial cells are renewed constantly and can remarkably proliferate and repair under normal conditions. Villus epithelial cells can repair an injury within 24 h to keep the villi in their normal histological structure. Therefore, determining the height and width of villus can be helpful in judging the damage degree of intestinal mucosa and its repair as well as proliferative ability (21). The intestinal mucosa of rats is damaged post-irradiation. RTP1 pretreatment can fight against the intestinal mucosa injury caused by radiation and promote the proliferation and repair ability of intestinal mucosa, which facilitates the structural and functional restoration of the intestine.

The pathogenesis of radiation-induced intestinal mucosal barrier injury is not very clear (22). With the fast proliferation rate of crypt epithelial cells, these cells are extremely sensitive to radiation. A certain dose of irradiation can decrease villus height, mucosal atrophy, mucosal surface ulcer formation and intestinal mucosa barrier damage. Thus, the direct damage of radiation on the crypt and the subsequent destruction on villus epithelial structures are the main reasons for abnormal intestinal mucosa morphological structures (5). RTP1 pretreatment can reduce the apoptosis and necrosis of the intestinal epithelial cells and promote the proliferation and repair of intestinal mucosa. These effects may be attributed to the inhibitory effect of RTP1 on radiation-induced intestinal epithelial apoptosis and intracellular reactive oxygen generation. This study suggests new ideas for seeking a radiation-protective substance with high efficiency and low toxicity, which is worthy of further investigation.
